# Acupuncture May Exert Its Therapeutic Effect through MicroRNA-339/Sirt2/NF*κ*B/FOXO1 Axis

**DOI:** 10.1155/2015/249013

**Published:** 2015-01-28

**Authors:** Jia-You Wang, Hui Li, Chun-Mei Ma, Jia-Lu Wang, Xin-Sheng Lai, Shu-Feng Zhou

**Affiliations:** ^1^Department of Human Anatomy, College of Fundamental Medical Sciences, Guangzhou University of Chinese Medicine, Guangzhou, Guangdong 510006, China; ^2^Department of Pharmaceutical Sciences, College of Pharmacy, University of South Florida, 12901 Bruce B. Downs Boulevard, MDC 30, Tampa, FL 33612, USA; ^3^Department of Acupuncture and Moxibustion, College of Acupuncture and Moxibustion, Guangzhou University of Chinese Medicine, Guangzhou, Guangdong 510006, China; ^4^Guizhou Provincial Key Laboratory for Regenerative Medicine, Stem Cell and Tissue Engineering Research Center and Sino-US Joint Laboratory for Medical Sciences, Guiyang Medical University, Guiyang, Guizhou 550004, China

## Abstract

Recently, we have found that a number of microRNAs (miRNAs) and proteins are involved in the response to acupuncture therapy in hypertensive rats. Our bioinformatics study suggests an association between these miRNAs and proteins, which include miR-339 and sirtuin 2 (Sirt2). In this paper, we aimed to investigate whether Sirt2 was a direct target of miR-339 in neurons. In human SH-SY5Y cells, the luciferase assay implied that Sirt2 was likely a target of miRNA-339. Overexpression of miR-339 downregulated Sirt2 expression, while knockdown of miR-339 upregulated Sirt2 expression in human SH-SY5Y cells and rat PC12 cells. In addition, overexpression of miR-399 increased the acetylation of nuclear factor-*κ*B (NF-*κ*B) and forkhead box protein O1 (FOXO1) in SH-SY5Y cells, which are known targets of Sirt2. Our findings demonstrate that miR-339 regulates Sirt2 in human and rat neurons. Since Sirt2 plays a critical role in multiple important cellular functions, our data imply that acupuncture may act through epigenetic changes and subsequent action on their targets, such as miRNA-339/Sirt2/NF-*κ*B/FOXO1 axis. Some physiological level changes of neurons after altering the miR-339 levels are needed to validate the suggested therapeutic role of miR-339/Sirt2/NF-*κ*B/FOXO1 axis in response to acupuncture therapy in the future work.

## 1. Introduction

MicroRNAs (miRNAs) is a large family of endogenous, small noncoding RNAs with 20–25 nucleotides that have emerged as key posttranscriptional regulators of gene expression in mammals, bacteria, and plants [1–4]. The discovery of miRNAs has revolutionized our comprehension of the regulation of gene expression. Based on miRBase version 21 released in June 2014 (http://www.mirbase.org/), there are 1,881 miRNA precursors and 2,588 mature miRNAs in humans. miRNAs are predicted to control the activity of more than 30% of human genes [5]. miRNAs are transcribed as ~70 nucleotide stem-loop precursors and subsequently processed by the cytoplasmic RNase-III type enzyme Dicer to generate ~22 nucleotide mature products which can target and modulate gene expression by inhibiting translation and/or inducing degradation of target mRNAs [4, 6, 7]. The mature miRNA is incorporated into a RNA-induced silencing complex (RISC), which recognizes target mRNAs through imperfect base pairing with the miRNA. miRNAs act as adaptors that employ a silencing complex to target mRNAs by selective base pairing, primarily in the 3′-untranslated region (3′-UTR). Target interaction does not require perfect complementarity between microRNA and mRNA sequences, although near-perfect base pairing in a small region in the 5′-end (positions 2–8) of the miRNA (sometimes termed “seed”) appears to be one of the key determinants of target recognition [4]. Gain and loss of function studies have indicated that miRNAs play a critical role in the regulation of all key biological functions such as development, cell proliferation, cell differentiation, and apoptosis [3, 4]. miRNAs are also associated with many human pathologies such as cancer, heart diseases, diabetes, inflammatory diseases, autoimmune diseases, and Alzheimer's disease [1, 8–17].

Acupuncture is the stimulation of specific acupoints along the skin of the body involving the application of penetration of thin needles [18]. In the past 40 years, the mechanistic studies of acupuncture mainly focus on (1) the neural pathways involved in inflammation, pain, and cardiovascular effects [19–22]; (2) the involvement of the connective tissues and their association with neural regulation [23, 24]; and (3) other molecular pathways that are involved in neural response to acupuncture. In recent years, the application of functional neuroimaging, microarray technology, proteomic analysis, and metabolomics analysis has significantly improved our understanding of the mechanism of acupuncture [25–28]. However, although there are a large number of mechanistic reports on the mechanism of acupuncture in the literature, we still have a poor knowledge on how acupuncture works [29].

Recent studies have revealed that a group of miRNAs are involved in the neural mechanism of acupuncture treatment in hypertensive rats [30]. However, the targets of acupuncture-regulated miRNAs are not fully identified and validated and it is unknown whether this is involved in acupuncture's effects. In this study, we investigated if the regulation of sirtuin 2 (Sirt2) was regulated by miR-339 in human and rat neurons.

## 2. Materials and Methods

### 2.1. Bioinformatics Studies and Selection of Sirt2 as a Potential Target of miR-339

Before starting the study, we performed the microarray analysis to screen the miRNAs (including miRNA-339) that responded to acupuncture therapy and conducted the proteomic study to investigate the proteins (including Sirt2) that responded to acupuncture therapy in hypertensive rats [30, 31]. To check whether acupuncture therapy exerts its therapeutic effect through epigenetic changes and subsequent action on their target, we conducted a bioinformatics study to predict the miRNAs that could regulate the genes that responded to acupuncture in hypertensive rats. MicroRNA.org (http://www.microrna.org/) was applied to predict the miRNAs. From these predicted miRNAs, we choose the miRNAs which were consistent with our previous study as candidate miRNAs for validation. The bioinformatics study showed that seven miRNAs, including miR-339, were predicted to regulate the human Sirt2 gene by the microRNA.org program.

### 2.2. Cell Line and Cell Cultures

As previously described, rat PC12 pheochromocytoma cells (ATCC, Manassas, VA, USA) were grown in RPMI 1640 medium containing 10% fetal bovine serum (HyClone) and 5% horse serum (Invitrogen). PC12 cells were induced to differentiate after 5-day treatment with NGF by growing cells in low serum medium (RPMI 1640 medium with 1% horse serum) with 100 ng/mL NGF (R&D Systems).

The human SH-SY5Y neuroblastoma cell line was provided (ATCC, Manassas, VA, USA). Cells were grown in Dulbecco's modified Eagle's medium (DMEM) supplemented with 2 mM L-glutamine, penicillin (20 units/mL), streptomycin (20 mg/mL), and 10% (vol/vol) heat-inactivated fetal calf serum (Life Technologies). Cells were maintained at 37°C in a saturated humidity atmosphere containing 95% air and 5% CO_2_.

### 2.3. Generation of Sirt2 Construct and Luciferase Assays

The* Sirt2 *3′-UTR target site was cloned into pMIR-reporter luciferase vector (Invitrogen) using the following oligonucleotides (Integrated DNA Technologies Inc., Coralville, Iowa, USA): sense, 5-CTAGTTTAACTCTTCCAGGACAGGGGGATCCA-3; and antisense, 5- AGCTTGGATCCCCCTGTCCTGGAAGAGTTAAA-3. We used a mutated sequence by inserting the following oligonucleotides (Integrated DNA Technologies Inc.): sense, 5- CTAGTTTAACTCTTCCACGCCCGAGGGATCCA-3; and antisense, 5- AGCTTGGATCCCTCGGGCGTGGAAGAGTTAAa-3.

SH-SY5Y cells were cultured in six-well plates at 1 × 10^5^ cells/well and transfected with different reporter vectors (300 ng Luc-Empty, 300 ng Luc-Sirt2-3′-UTR, or 300 ng Luc-Sirt2-3′-UTR-mut) and cotransfected with different concentration of miRNA-339 mimic (10 and 50 nM). Cells were assayed 48 h after transfection with the dual-luciferase reporter assay system (Invitrogen). Luciferase activity was normalized by *β*-galactosidase activity.

### 2.4. Overexpression and Knockdown of miR-339 via Transfection

The nontargeting miRNA control, miR-339 mimic, and miR-339 inhibitor were obtained from Invitrogen. Cells were seeded in six-well plates at 1 × 10^5^ cells/well and transfected with different concentration of miRNA-339 mimic, miRNA-339 inhibitor, or nontargeting miRNA control (10 and 50 nM) using Lipofectamine 2000 (Invitrogen) according to the manufacturer's protocol. Then, the cells were collected for further analysis.

### 2.5. Western Blotting Assay

The cells were harvested and lysed with the lysis buffer (50 mmol HEPES at pH 7.5, 150 mmol NaCl, 10% glycerol, 1.5 mmol MgCl_2_, 1% Triton-X 100, 1 mmol EDTA at pH 8.0, 10 mmol sodium pyrophosphate, 10 mmol sodium fluoride, and the protease inhibitor cocktail) and centrifuged at 12,000 g for 15 min at 4°C. Proteins in cell lysates (30 *μ*g/lane) were separated by 10% SDS-PAGE, blotted, and detected with antibodies against Sirt2 (1 : 1000; Cell Signaling Technology Inc.), acetyl-NF*κ*B p65 (1 : 1000; Cell Signaling Technology Inc.), and acetylated Foxo1 (AC-FKHR; 1 : 200; Santa Cruz Biotechnology, Inc., Texas, USA). Protein level was normalized to the matching densitometric value of the internal control *β*-actin (1 : 1000, Cell Signaling Technology Inc.).

### 2.6. Statistical Analysis

Data are presented as the mean ± S.D. Multiple comparisons were evaluated by one-way analysis of variance (ANOVA) followed by Tukey's multiple comparison procedure, with *P* < 0.05 considered significant. All statistical tests were performed using Prism software version 6.0 (GraphPad Software Inc.).

## 3. Results

### 3.1. Luciferase Assay Implies That Sirt2 Is Likely a Direct Target of miRNA-339 in SH-SY5Y Cells

**Figure 1:**
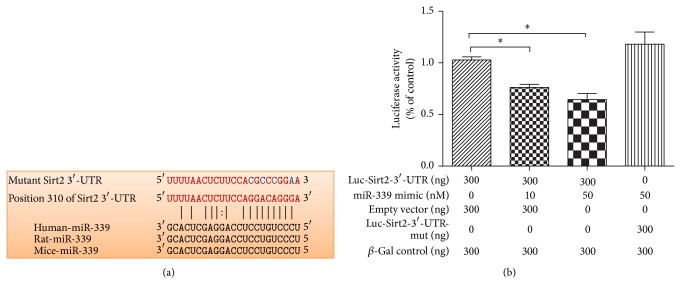
Sirt2 is regulated by miR-339 directly. (a) Sequence of human miR-339 and the predicted binding sites with miR-339 within Sirt2 untranslated region (3′-UTR) from different species are shown. The sequence of Sirt2 3′-UTR mutant used for reporter assay is also shown. (b) Luciferase reporter constructs containing 3′-UTR (Luc-Sirt2-3′-UTR) or mutant 3′-UTR (Luc-Sirt2-3′-UTR-mut) of* Sirt2* gene were cotransfected with miR-339 mimic or empty vector in SH-SY5Y cell line and the luciferase activities were assayed (*n* = 6, ^*^ *P* < 0.05 versus control).

To validate whether Sirt2 is a likely target of miR-339, we performed computational miRNA target analysis, which showed that miRNA-339 was able to bind to the Sirt2 mRNA 3′-UTR, suggesting this gene might be a potential target for miRNA-339 (Figure 1(a)). Moreover, to examine whether miR-339 could repress Sirt2 expression through direct 3′-UTR interaction, we cloned Sirt2 3′-UTR luciferase reporter plasmid and performed reporter analysis in SH-SY5Y cells. Our present data demonstrated that cotransfection of miR-339 mimic with Sirt2 3′-UTR reporter resulted in dose-dependent inhibition of luciferase activity in SH-SY5Y cells (Figure 1(b), *P* < 0.05 versus control). However, miR-339 failed to repress the activity of Sirt2-3′-UTR reporter with a mutated miR-339 seed sequence (Figure 1(b)). These data indicated that Sirt2 was likely a direct target of miR-339.

### 3.2. Overexpression of miRNA-339 Downregulates Sirt2 Expression in Human SH-SY5Y Cells

**Figure 2:**
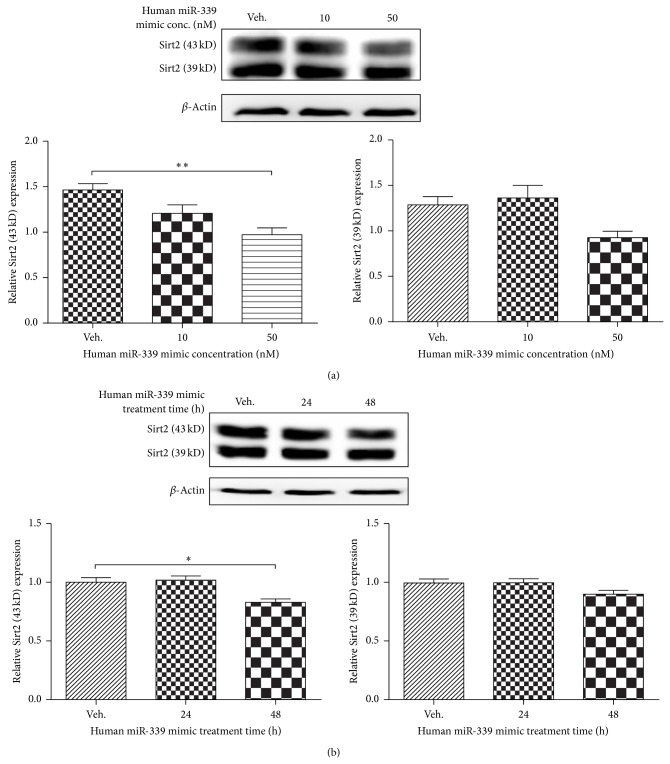
Overexpression of miRNA-339 inhibited Sirt2 expression in human neurons. (a) Overexpression of miR-339 inhibited Sirt2 expression in a concentration-dependent manner. SH-SY5Y cells were treated with miRNA-339 mimic at 10 and 50 nM. Forty-eight hours later, the cell lysates were collected for western blotting. *N* = 6, ^**^ *P* < 0.01 versus control. (b) Overexpression of miR-339 inhibited Sirt2 expression in a time-dependent manner. SH-SY5Y cells were treated with miRNA-339 mimic at 50 nM for 24 and 48 h. At each time point, the cell lysates were collected for western blotting. *n* = 6, ^*^ *P* < 0.05 versus control.

To further validate whether miRNA-339 could downregulate Sirt2 expression in human neurons, we tested the effect of miRNA-339 on Sirt2 expression level in SH-SY5Y cells transfected with miRNA-339 mimic. Overexpression of miRNA-339 significantly decreased Sirt2 expression in a dose-dependent (Figure 2(a), *P* < 0.01 versus control) and time-dependent manner (Figure 2(b), *P* < 0.05 versus control).

### 3.3. Overexpression of miRNA-339 Downregulates Sirt2 Expression in Rat PC12 Cells

**Figure 3:**
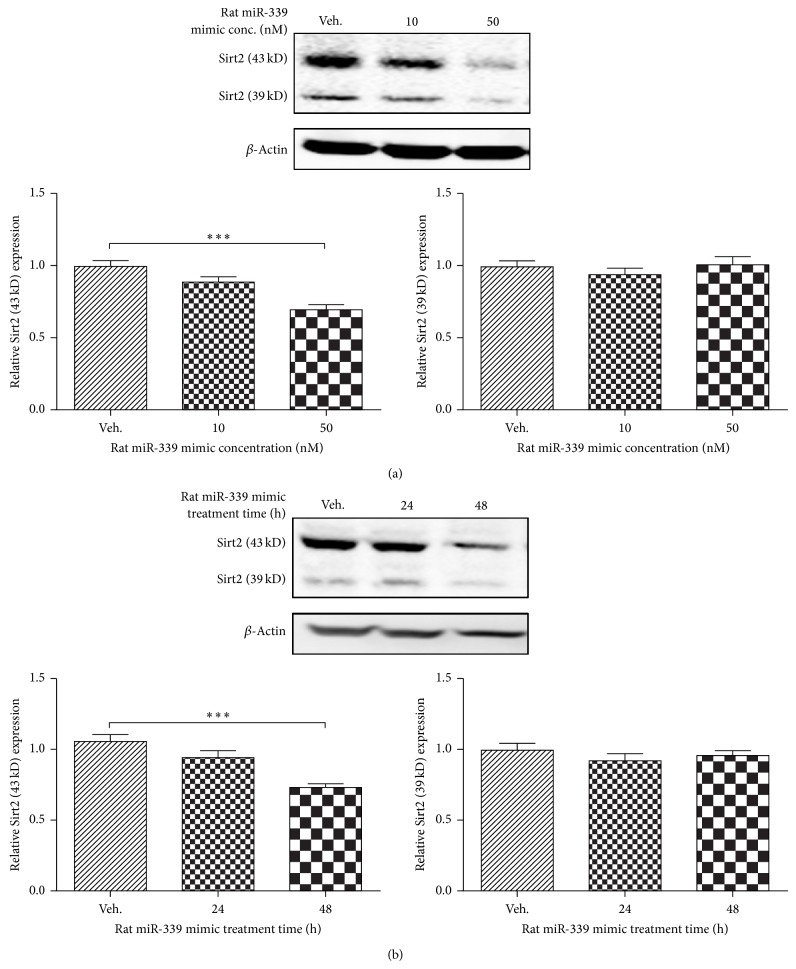
Overexpression of miRNA-339 also inhibited Sirt2 expression in rat neurons. (a) Overexpression of miR-339 inhibited Sirt2 expression in a concentration-dependent manner. NGF-induced differentiated PC12 cells were treated with miRNA-339 mimic at 10 and 50 nM. Forty-eight hours later, the cell lysates were collected for western blotting. *n* = 6, ^***^ *P* < 0.001 versus control. (b) Overexpression of miR-339 inhibited Sirt2 expression in a time-dependent manner. NGF-differentiated PC12 cells were treated with miRNA-339 mimic at 50 nM for 24 and 48 h. At each time point, the cell lysates were collected for western blotting. *n* = 6, ^***^ *P* < 0.001 versus control.

To further validate whether miRNA-339 can downregulate Sirt2 expression in rat neurons, we tested the effect of miRNA-339 on Sirt2 expression level in NGF-induced PC12 cells transfected with miRNA-339 mimic. Overexpression of miRNA-339 significantly decreased Sirt2 expression in a dose-dependent (Figure 3(a), *P* < 0.01 versus control) and time-dependent manner (Figure 3(b), *P* < 0.001 versus control).

### 3.4. Knockdown of miRNA-339 Upregulates Sirt2 Expression in Rat PC12 Cells

**Figure 4:**
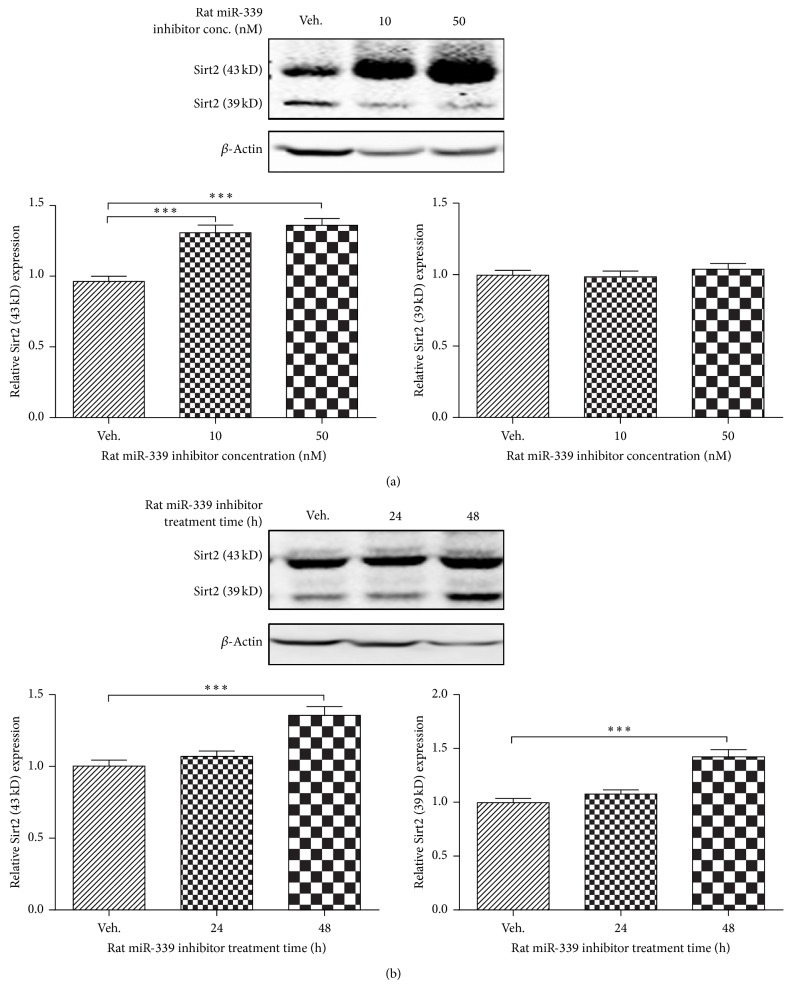
Knockdown of miRNA-339 increased Sirt2 expression in rat neurons. (a) Knockdown of miR-339 increased Sirt2 expression in a concentration-dependent manner. NGF-induced differentiated PC12 cells were treated with miRNA-339 inhibitor at 10 and 50 nM. Forty-eight hours later, the cell lysates were collected for western blotting. *n* = 6, ^***^ *P* < 0.001 versus control. (b) Knockdown of miR-339 increased Sirt2 expression in a time-dependent manner. NGF-differentiated PC12 cells were treated with miRNA-339 inhibitor at 50 nM for 24 and 48 h. At each time point, the cell lysates were collected for western blotting. *n* = 6, ^***^ *P* < 0.001 versus control.

To further validate whether knockdown of miRNA-339 can upregulate Sirt2 expression in rat neurons, we tested the effect of miRNA-339 on Sirt2 expression level in NGF-induced PC12 cells transfected with miRNA-339 inhibitor. Knockdown of miRNA-339 significantly increased Sirt2 expression in a dose-dependent (Figure 4(a), *P* < 0.01 versus control) and time-dependent manner (Figure 4(b), *P* < 0.001 versus control).

### 3.5. Overexpression of miRNA-339 Upregulates the Acetylated NF-*κ*B and FOXO1 Expression Level in SH-SY5Y Cells

**Figure 5:**
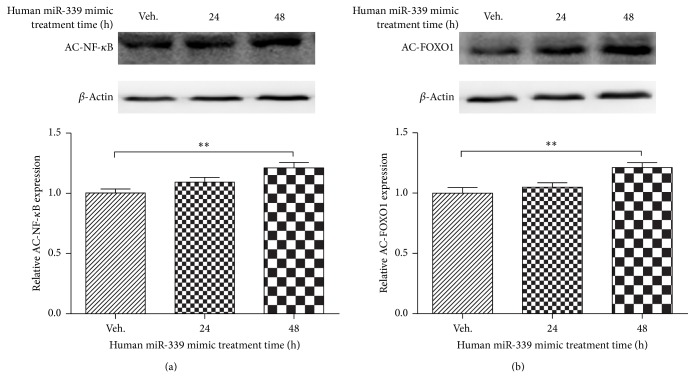
MiRNA-339 increased the acetylated NF-*κ*B and FOXO1 expression level. Overexpression of miRNA-339 significantly increased the acetylated NF-*κ*B (a) and FOXO1 (b) level in a time-dependent manner. SH-SY5Y cells were treated with miRNA-339 mimic at 50 nM for 24 and 48 h. At each time point, the cell lysates were collected for western blotting. *n* = 6, ^**^ *P* < 0.01 versus control.

Our results showed that Sirt2 is the direct target of miR-339, indicating that miR-339 can contribute to an upregulation of the acetylated status of Sirt2 target, including NF-*κ*B and FOXO1. To test this hypothesis, we measured the acetylated status of Sirt2 target (NF-*κ*B and FOXO1) in SH-SY5Y cells treated with miRNA-339 mimic. As we expected, overexpression of miRNA-339 significantly increased the acetylation level of NF-*κ*B and FOXO1 in a time-dependent manner (Figures 5(a) and 5(b), *P* < 0.01 versus control).

## 4. Discussion

In this paper, we firstly found that one of the acupuncture-regulated targets, Sirt2, is likely a direct target of acupuncture-regulated miRNA-339 in neurons. In addition, we demonstrated that altered Sirt2 expression by miR-339 activates its targets such as NF-*κ*B and FOXO1 through increasing their acetylation. Our findings implied that acupuncture may act through epigenetic changes and subsequent action on their targets, such as miRNA-339/Sirt2/NF-*κ*B/FOXO1 axis.

Sirt2 is one of the sirtuin family (SIRT1–7), which is known to be most predominantly expressed in the brain. Sirtuins are a class of proteins that possess deacylase activity, including deacetylase activity [32–36]. Sirtuins have been implicated as a regulator of a variety of important biological process, like aging, metabolism, and stress resistance [34]. Our previous study and other studies have showed that acupuncture can exert its therapeutic effect involved with regulating sirtuin expression (including Sirt1 and Sirt2) [31, 35, 37]. Our present data showed that Sirt2 is likely a direct target of miRNA-339, which leads to a better explanation as to why acupuncture upregulated miRNA-339 expression companion with acupuncture downregulation of Sirt2 expression in hypertensive rats. There are three transcript isoforms of Sirt2 gene, including isoform 1 (43 kD), isoform 2 (39 kD), and isoform 5, but only 43 kD Sirt2 was affected after miR-339 overexpression or knockdown according to our present data. One of the possible explanations is that the human Sirt2 mRNA has an open reading frame of 1,167 bp that encodes two isoforms of the Sirt2 protein: isoform 1 encodes a 389-aa protein with a predictive molecular weight of 43.2 kD, while isoform 2, which is lacking the first three exons, encodes a 352-aa protein with a predictive molecular weight of 39.5 kD. Our data also showed that miR-339 increased the acetylated NF-*κ*B and FOXO1 expression level. NF-*κ*B and FOXO1 are targets of Sirt2. Decreased expression level of Sirt2 activates NF-*κ*B and FOXO1 by increasing its acetylation. This could lead to exerting acupuncture therapeutic effect. Further studies are needed to validate whether miR-339 increased the acetylated NF*κ*B and FOXO1 by regulating the protein levels, stability, or activity of Sirt2.

**Figure 6:**
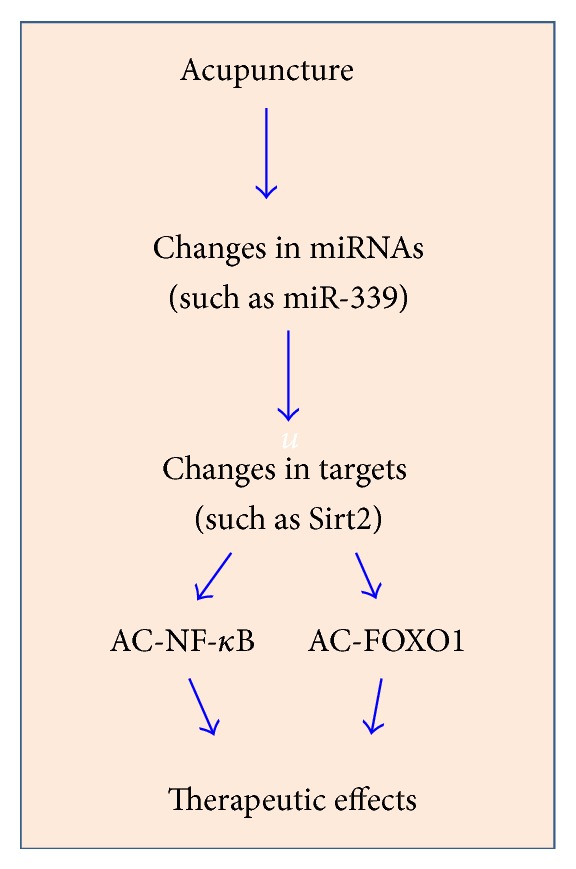
Proposed mechanisms of the therapeutic effect of acupuncture through epigenetic pathways. We propose that acupuncture exerts its therapeutic effect through regulation of miRNAs and its targets' axis, such as miRNA-339/Sirt2/NF-*κ*B/FOXO1 axis.

Taken together, we proposed that acupuncture exerts its therapeutic effects through a series of biological processes including (Figure 6) (1) changes of miRNAs (such as miR-339); (2) changes of targets (such as Sirt2); and (3) altered expression levels of Sirt2 activating its targets such as NF*κ*B and FOXO1. These events will result in remarkable therapeutic effects observed in vivo.

A possible limitation of the present study was that some physiological level changes of neurons after altering the miR-339 levels are needed to validate the proposed therapeutic role of miR-339/Sirt2/NF*κ*B/FOXO1 axis in response to acupuncture therapy in the future work.
